# Successful Percutaneous Endovascular Repair in a Very Large, Symptomatic, Non-ruptured Abdominal Aortic Aneurysm with Severe Neck Angulation

**DOI:** 10.7759/cureus.5125

**Published:** 2019-07-11

**Authors:** Bradley M White, Dustin L Meyer, Aswin Kumar, Curtis L Anderson

**Affiliations:** 1 Interventional and Diagnostic Radiology, Larkin Community Hospital, Miami, USA; 2 Radiology, University of South Florida Morsani College of Medicine, Tampa, USA; 3 Interventional Radiology, Florida Endovascular and Interventional, Miami Lakes, USA

**Keywords:** endovascular aneurysm repair (evar), percutaneous endovascular aneurysm repair (pevar), abdominal aortic aneurysm (aaa), hostile neck, severe neck angulation, impending rupture

## Abstract

Percutaneous endovascular aneurysm repair (PEVAR) is a minimally invasive treatment option for patients with abdominal aortic aneurysms (AAA). PEVAR allows for the lower incidence of vascular access site complications and decreased procedure time, yet the utility of this technique depends on the anatomical characteristics of the aneurysm. A detailed evaluation of the access site vessels and aneurysm neck anatomy are critical for standard patient and device selection.

An 84-year-old male presented to our institution with the sudden onset of abdominal pain and confusion. Subsequent imaging demonstrated the presence of a 9.5 cm fusiform, infrarenal abdominal aortic aneurysm with a greater than 60-degree neck angulation and bilateral common iliac aneurysms. The patient underwent percutaneous endovascular aneurysm repair (PEVAR), and a type IB endoleak seen at the end of the case was treated successfully. At the one-year follow-up, the patient remained asymptomatic with the AAA stable in size.

This case represents the largest reported symptomatic unruptured AAA repaired with a completely percutaneous technique to date. Building up the stent-graft from the bifurcation with a unibody modular device may allow for support to address the severe angulation of a very hostile neck. PEVAR is a viable option in patients with symptomatic AAA and can be performed despite severe aneurysm neck angulation.

## Introduction

Abdominal aortic aneurysm (AAA) is defined as a focal diameter > 3.0 cm [1]. It is a common manifestation of vascular disease in older adults. Endovascular aneurysm repair (EVAR) is a minimally invasive procedure that has historically demonstrated lower morbidity and mortality compared to open surgical repair [2]. The feasibility of EVAR depends on the anatomical characteristics of the aneurysm. The access site vessel tortuosity, the diameter of the aneurysm, and the aneurysm neck are prudent pre-procedural considerations. Characteristics of an aneurysm that increase complications during repair include a diameter > 30 mm, neck length < 15 mm, and neck angulation > 60 degrees [3]. The aneurysm neck angle is the most important factor in a successful EVAR because this seal zone is crucial to prevent endoleak and distal migration of the stent graft [3-5].

The modern innovations in vascular devices (to include stent grafts) continue to allow skilled interventionalists to successfully treat increasingly complex aneurysms. Our case report describes the successful repair of an infrarenal AAA with a 9.5 cm transverse diameter and a 90-degree neck angulation.

## Case presentation

**Figure 1:**
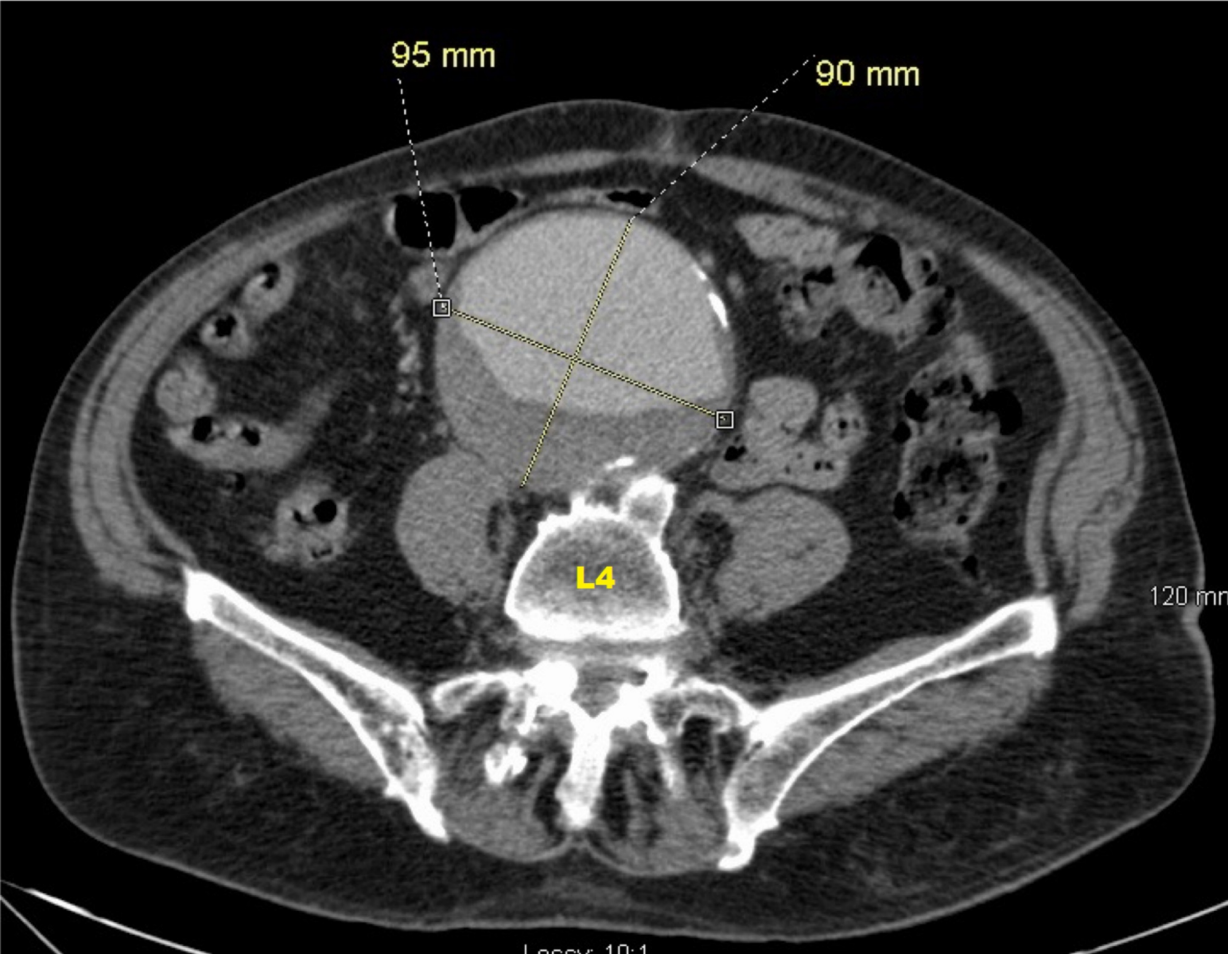
Contrast-enhanced axial CT of the abdomen and pelvis (level of L4) image showing 9.5 x 9.0 cm infrarenal AAA with peripheral mural thrombus AAA: abdominal aortic aneurysm; CT: computed tomography

**Figure 2:**
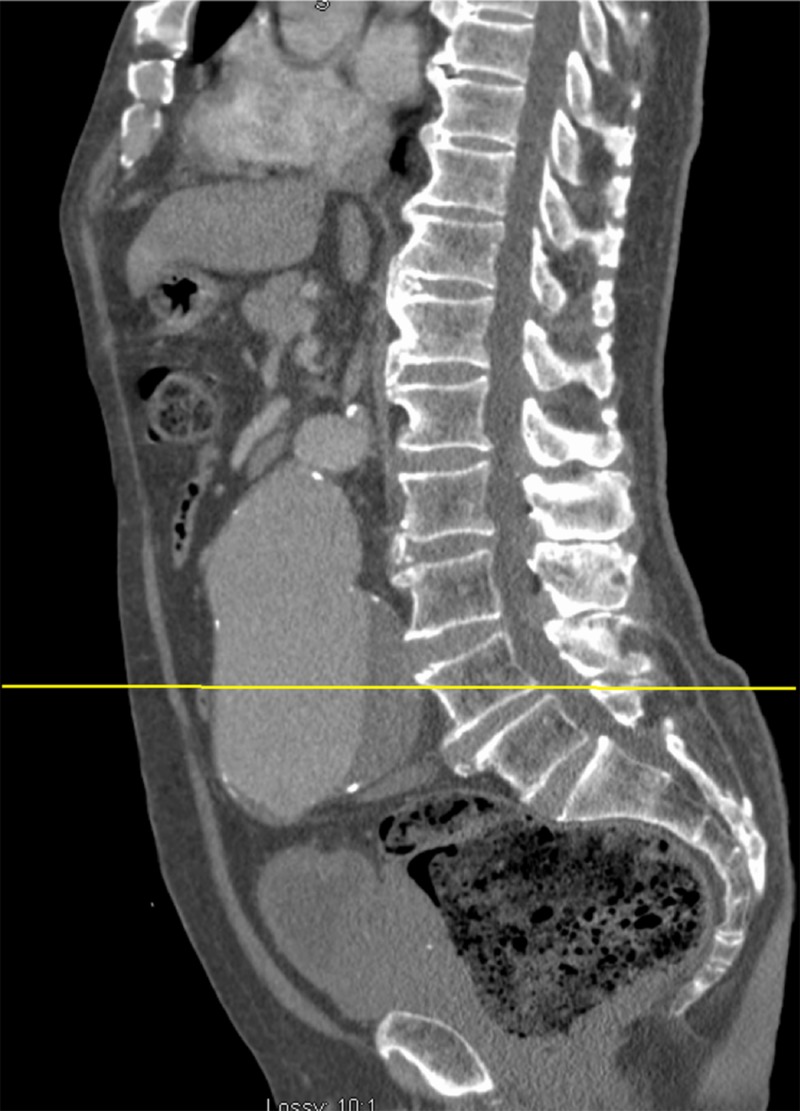
Contrast-enhanced midline sagittal CT image showing a 9.5 cm infrarenal AAA with mural thrombus The yellow horizontal line denotes the level of the axial image in Figure 1. AAA: abdominal aortic aneurysm; CT: computed tomography

**Figure 3:**
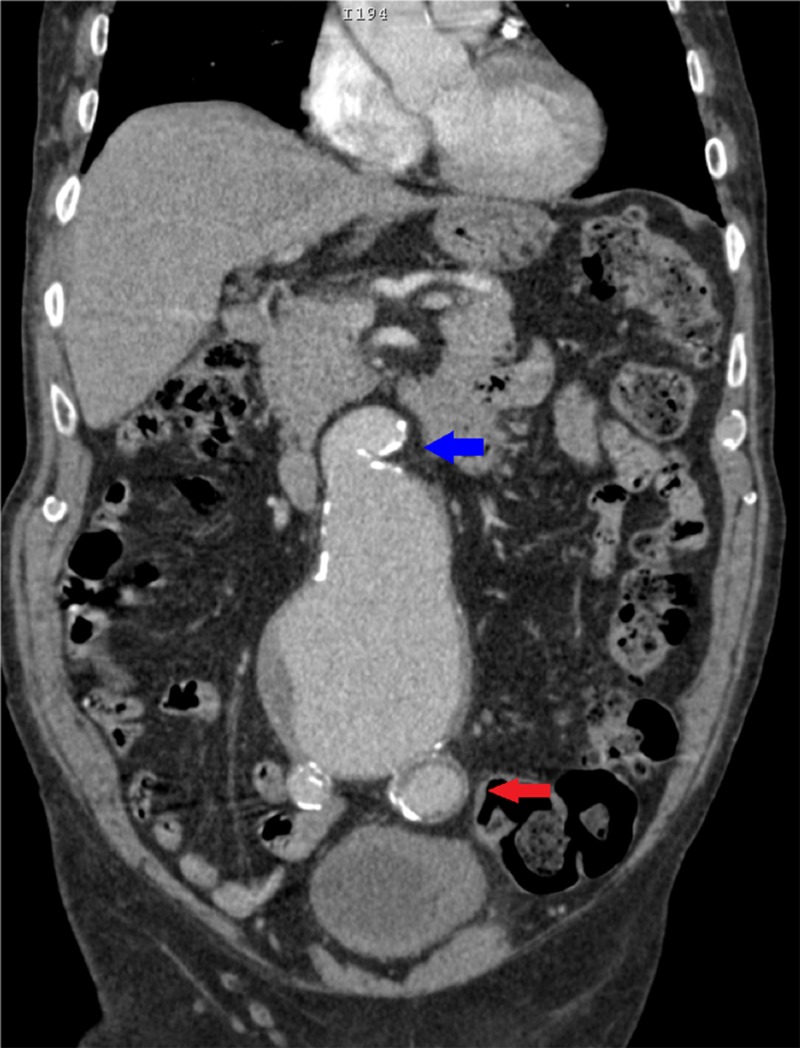
Coronal view of a contrast-enhanced CT of the abdomen and pelvis showing a 9.5 cm infrarenal AAA with a 90-degree proximal neck angulation (blue arrow) and peripheral mural thrombus. Additionally, there is also a 3.3 cm left common iliac artery aneurysm (red arrow). AAA: abdominal aortic aneurysm; CT: computed tomography

Our patient is an obese 84-year-old Hispanic male who initially presented to our Emergency Department as a transfer from an assisted living facility after sustaining a ground-level mechanical fall. Following a benign head and neck imaging workup, the patient voiced concerns of generalized abdominal discomfort. Subsequent diagnostic contrast-enhanced computed tomography (CT) of the abdomen and pelvis were performed which identified a 9.5 cm fusiform infrarenal abdominal aortic aneurysm. The unique anatomical characteristics of the aneurysm included a maximum transverse diameter of 9.5 cm, a 90-degree neck angulation, and a 3.1 cm proximal neck width (Figures 1-3). Additionally, there were accompanying aneurysms of the left common iliac and left internal iliac arteries (Figure 3). The patient was admitted under the care of the interventional radiology service. Due to the patient’s increased risk of rupture secondary to symptomatic presentation and size of the aneurysm, the patient was scheduled for emergent repair [6-7].

Procedure technique

**Figure 4:**
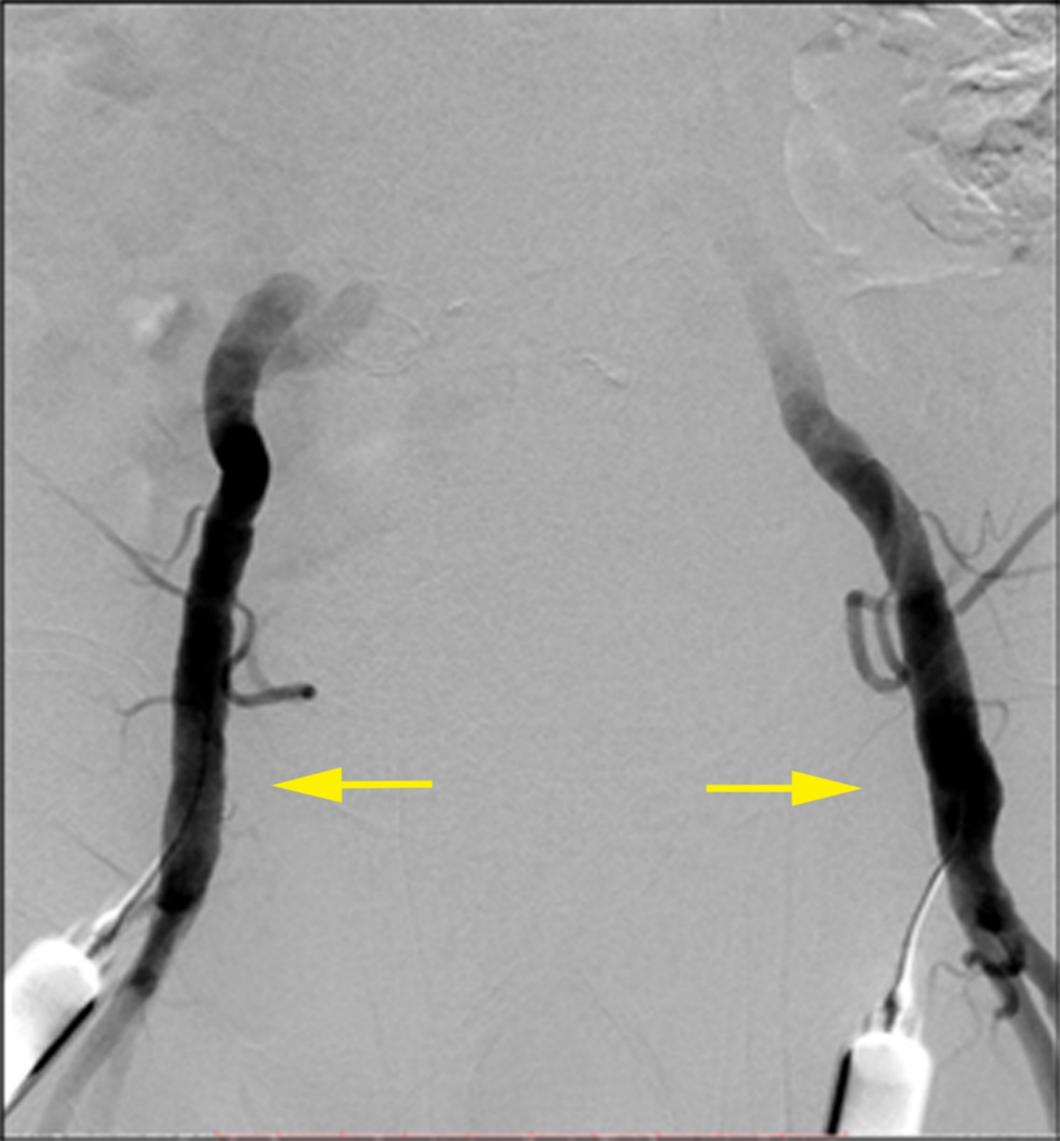
Bilateral common femoral artery percutaneous access (yellow arrows) on anteroposterior digital subtraction angiography (DSA).

**Figure 5:**
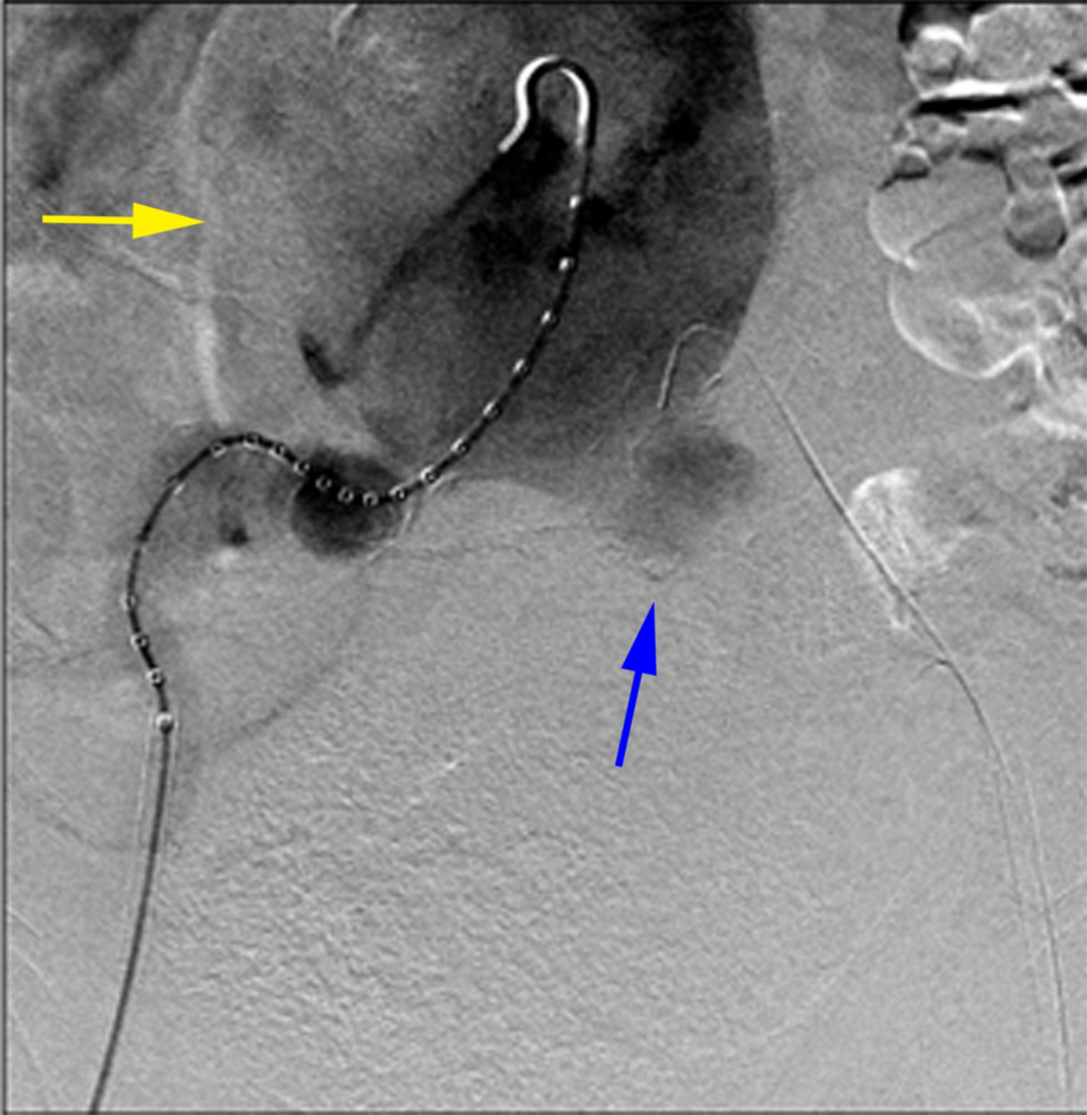
Anteroposterior digital subtraction angiogram of the abdominal aorta demonstrated a very large infrarenal abdominal aortic aneurysm (yellow arrow) with a left common iliac artery aneurysm (blue arrow).

**Figure 6:**
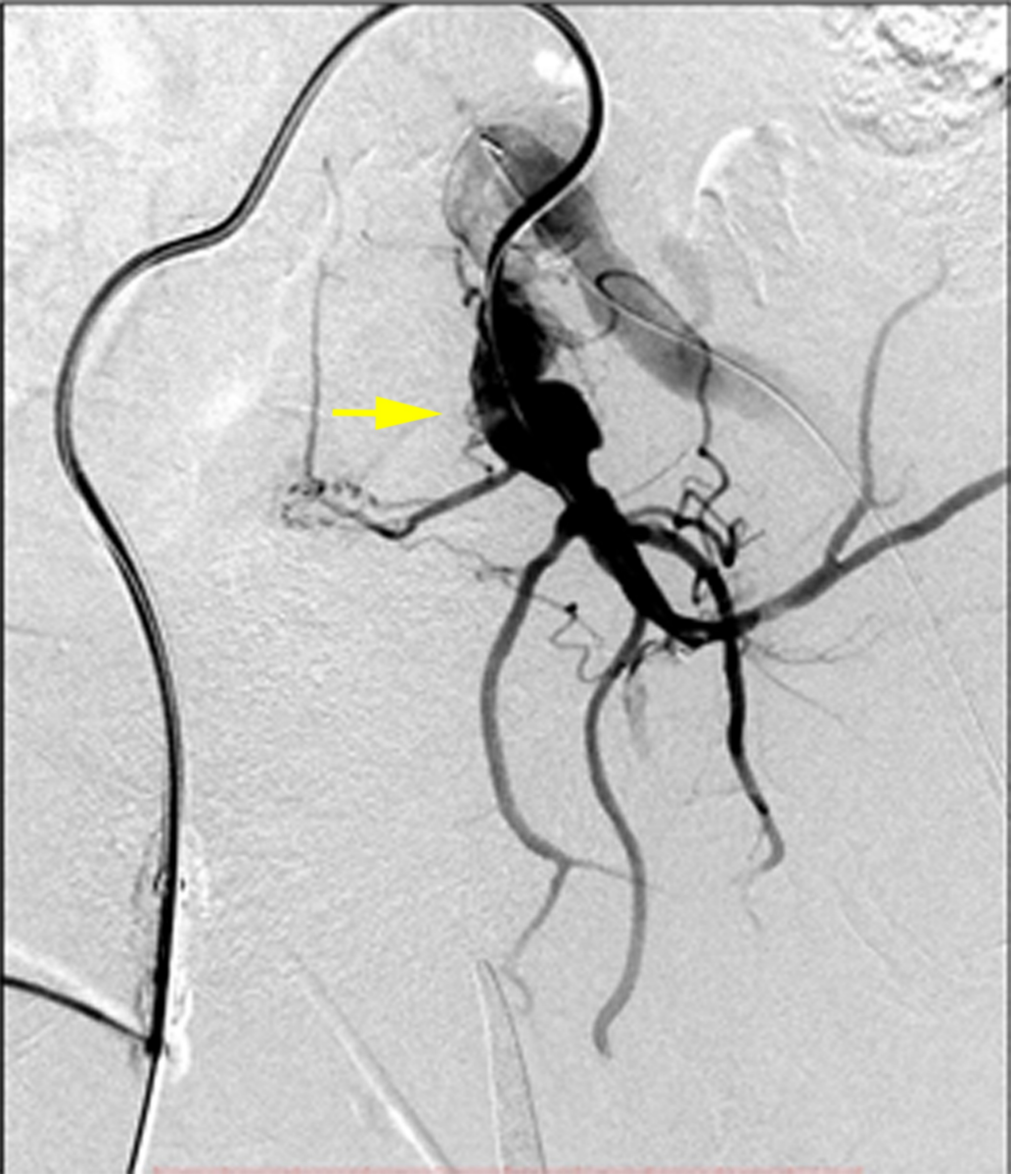
Selective RAO DSA demonstrating a left internal iliac artery aneurysm (yellow arrow) DSA: digital subtraction angiogram; RAO: right anterior oblique

**Figure 7:**
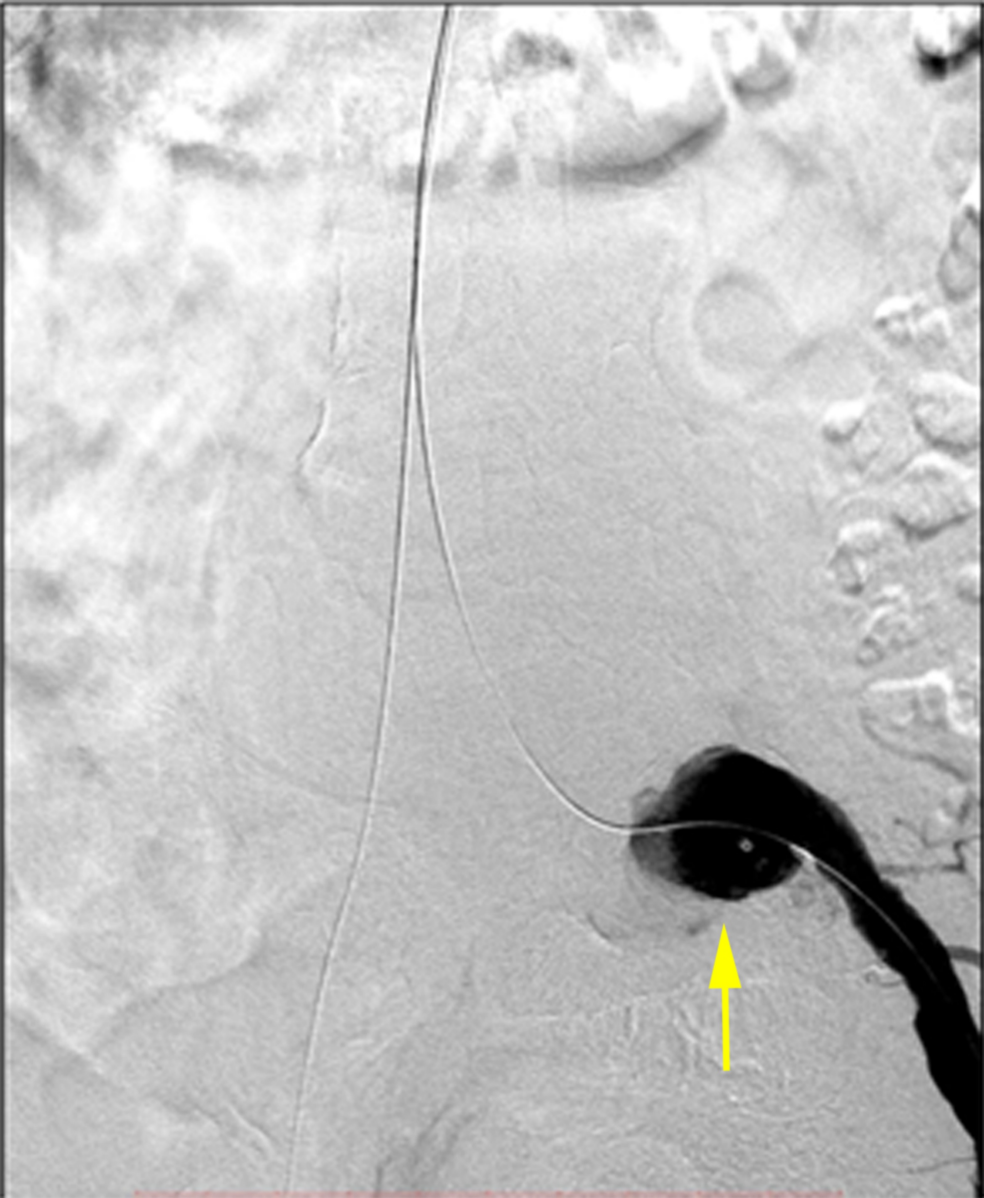
The left internal iliac artery was embolized using an 18 mm AVP II. The remaining aneurysm of the left common iliac artery is demonstrated (yellow arrow).

On the following morning, the patient underwent percutaneous endovascular aortic aneurysm repair (PEVAR). The procedure was performed under general anesthesia. It was initiated with ultrasound-guided bilateral femoral access, dilation, and sheath placement (Figure 4). Four sutures (Perclose Pro-Glide SMC; Abbott, Santa Clara, CA) were delivered percutaneously in a “pre-close” fashion. Using a multipurpose angiographic (MPA) catheter and guide-wire, the abdominal aorta was catheterized and an aortogram was performed (Figure 5). Next, the left internal iliac artery selective arteriogram demonstrated an aneurysm (Figure 6). A 7-French 45-cm sheath was advanced through the right groin into the left internal iliac artery. The left internal iliac artery aneurysm was embolized using an AMPLATZER vascular plug (18-mm AVP II; AGA Medical Corp, Golden Valley, MN) (Figure 7).

**Figure 8:**
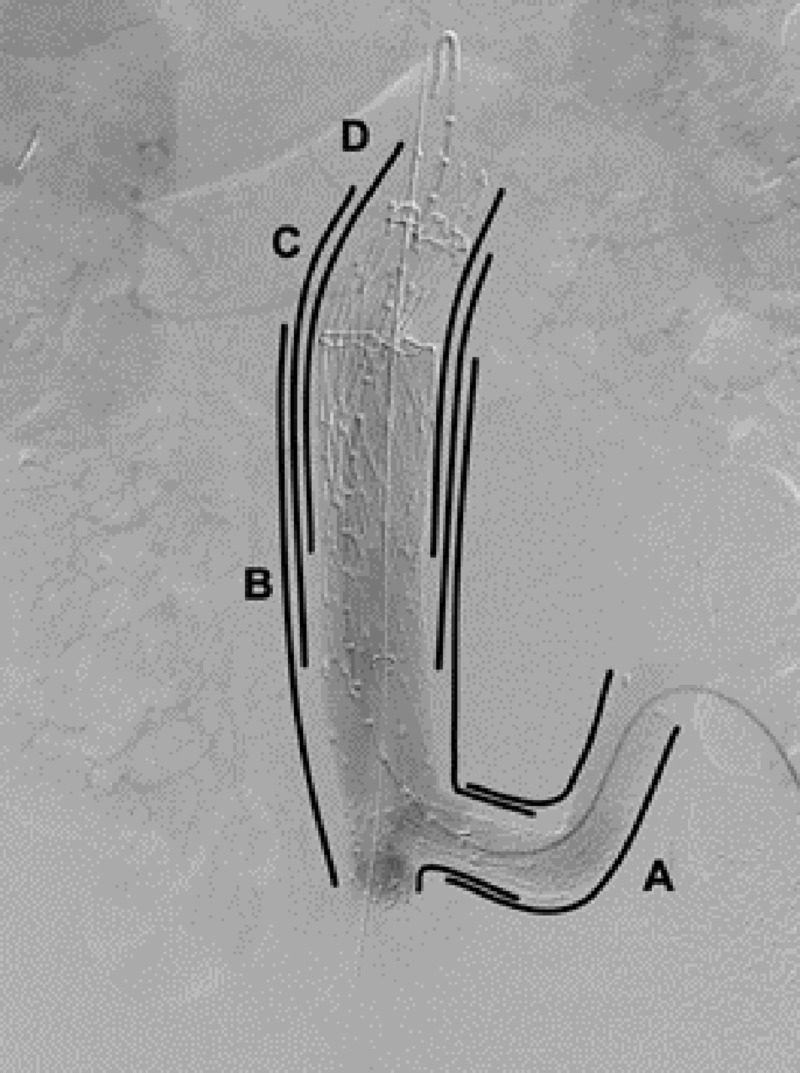
Sequential procedural diagram, anteroposterior DSA: (A) Ovation iliac limb; (B) Main body deployed down to the aortic bifurcation; (C) Cuff extension deployed superiorly; (D) Final cuff deployed to the origin of the renal arteries DSA: digital subtraction angiogram

A less hostile distal landing zone was created by deploying a 14 mm x 100 mm Ovation iX™ iliac limb (TriVascular, Inc., Santa Rosa, CA) into the left common iliac artery (Figure 8A). Next, working through the left groin, an MPA catheter and glide-wire were used to catheterize the abdominal aorta. A Lunderquist® wire (Cook Medical, Bloomington, IN, CA) was advanced into the thoracic aorta. Subsequently, a sheath was advanced into the right groin, through which a 25 mm x 100 mm AFX® unibody main body segment (Endologix, Inc., Irvine, CA) was advanced. The contralateral limb was snared and the device was deployed and pulled down to the aortic bifurcation (Figure 8B). The left iliac limb of the main body was deployed into the previously placed left limb extension.

**Figure 9:**
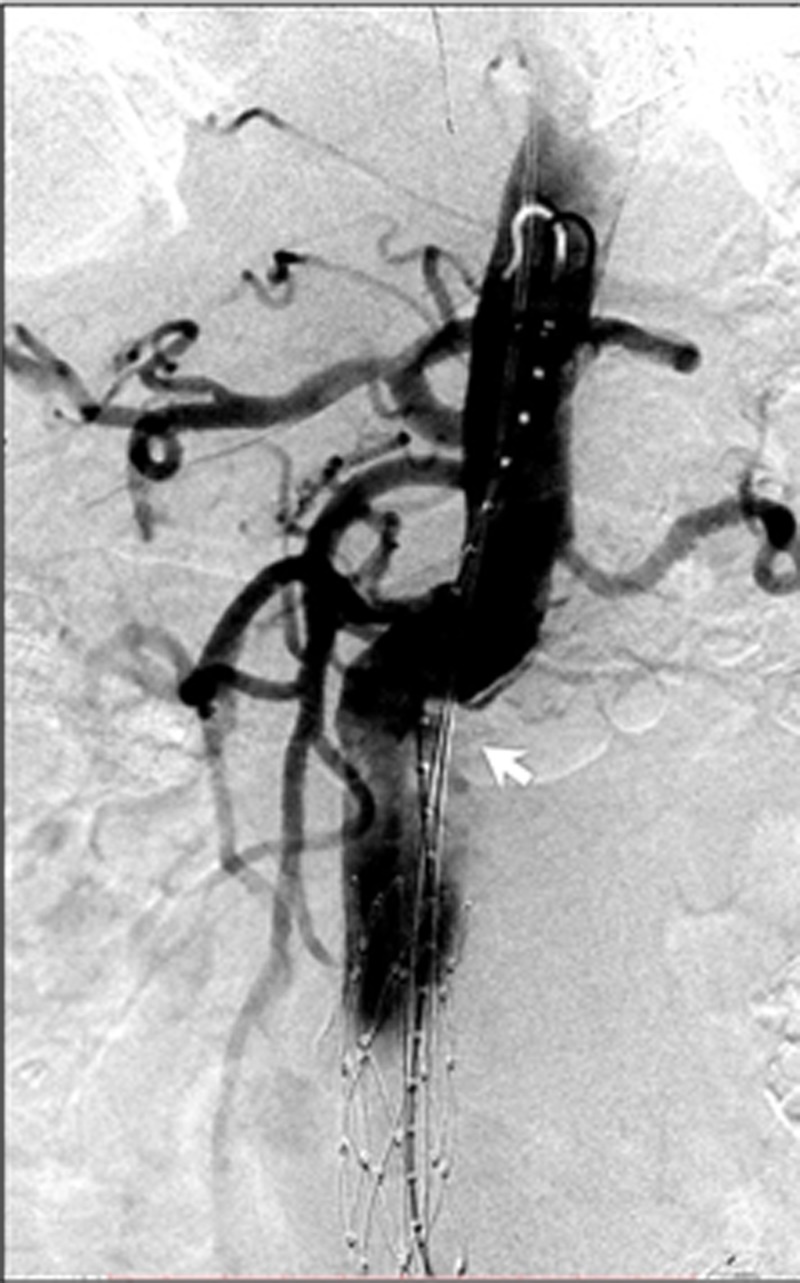
Severe (90-degree) proximal neck angulation (white arrow)

To correct the severe proximal neck angle (Figure 9), a 28 mm x 95 mm VELA™ infrarenal cuff (Endologix, Inc., Irvine, CA) was deployed through the right groin sheath and extended superiorly towards the angled neck (Figure 8C). A second and final 34 mm x 100 mm VELA suprarenal proximal cuff was deployed at the infrarenal aorta after marking the origin of the renal arteries using an aortogram (Figure 8D). Both of the proximal cuffs overlapped with the distal stent graft segment by 8 cm in order to avoid a possible type III endoleak.

**Figure 10:**
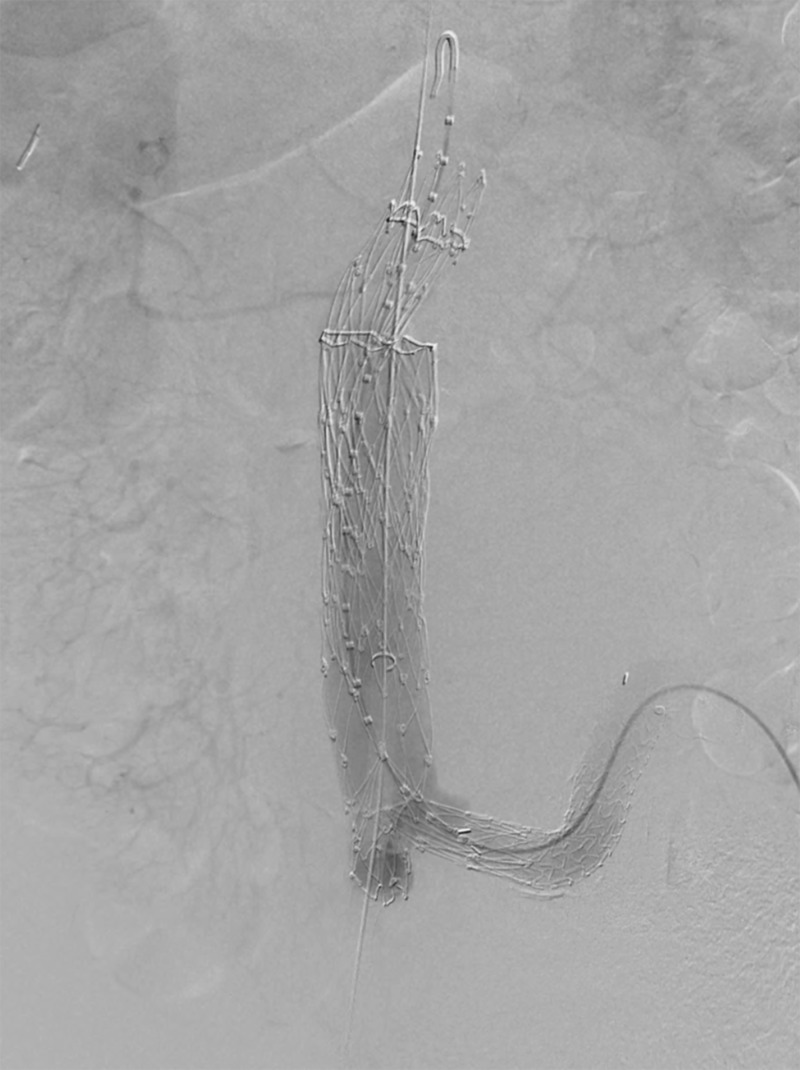
Anteroposterior digital subtraction angiogram showing a stent graft of the iliac limb, main body, and proximal cuffs

**Figure 11:**
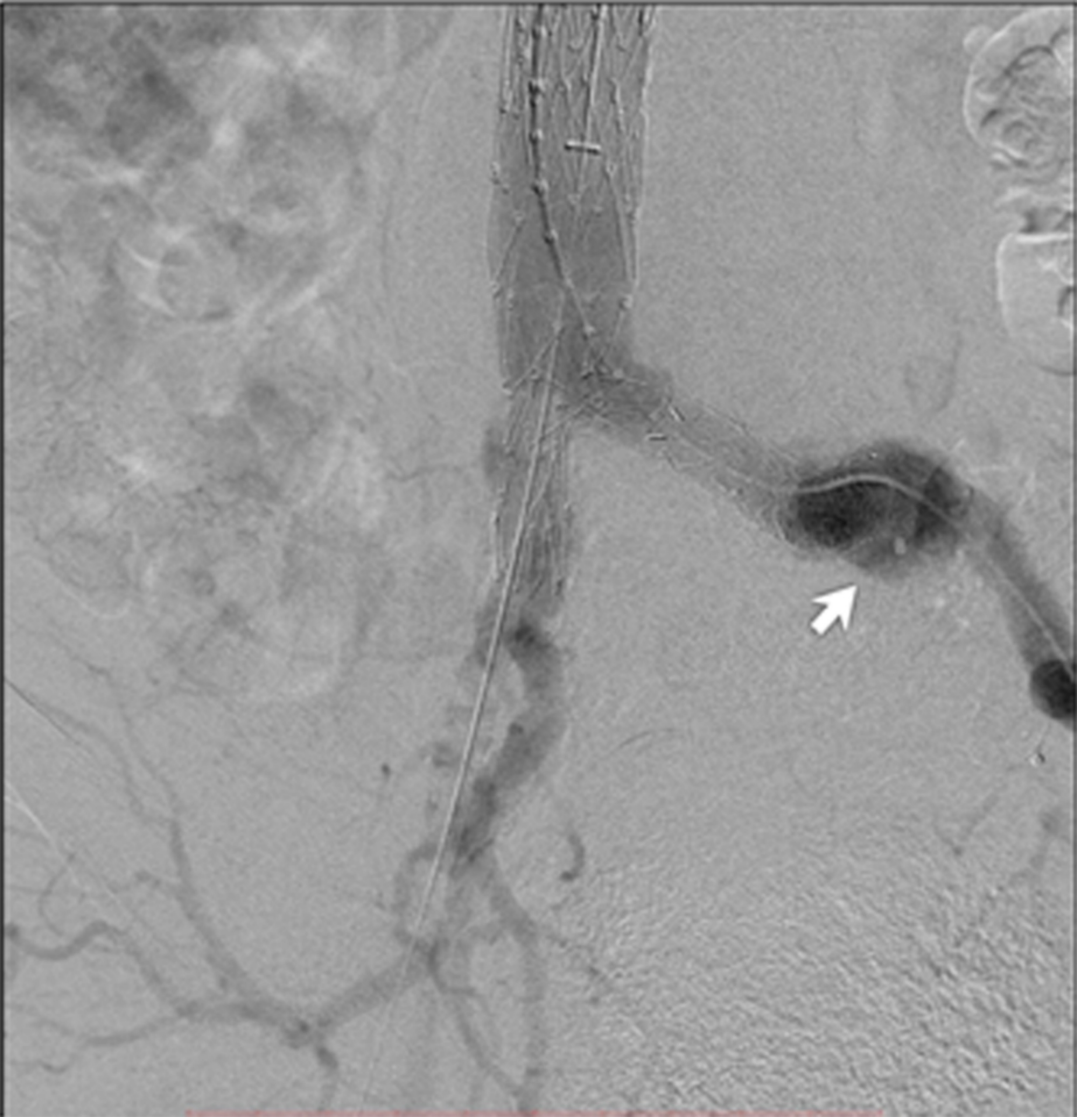
Anteroposterior digital subtraction angiogram demonstrating a type IB endoleak in the distal left common iliac artery (white arrow)

**Figure 12:**
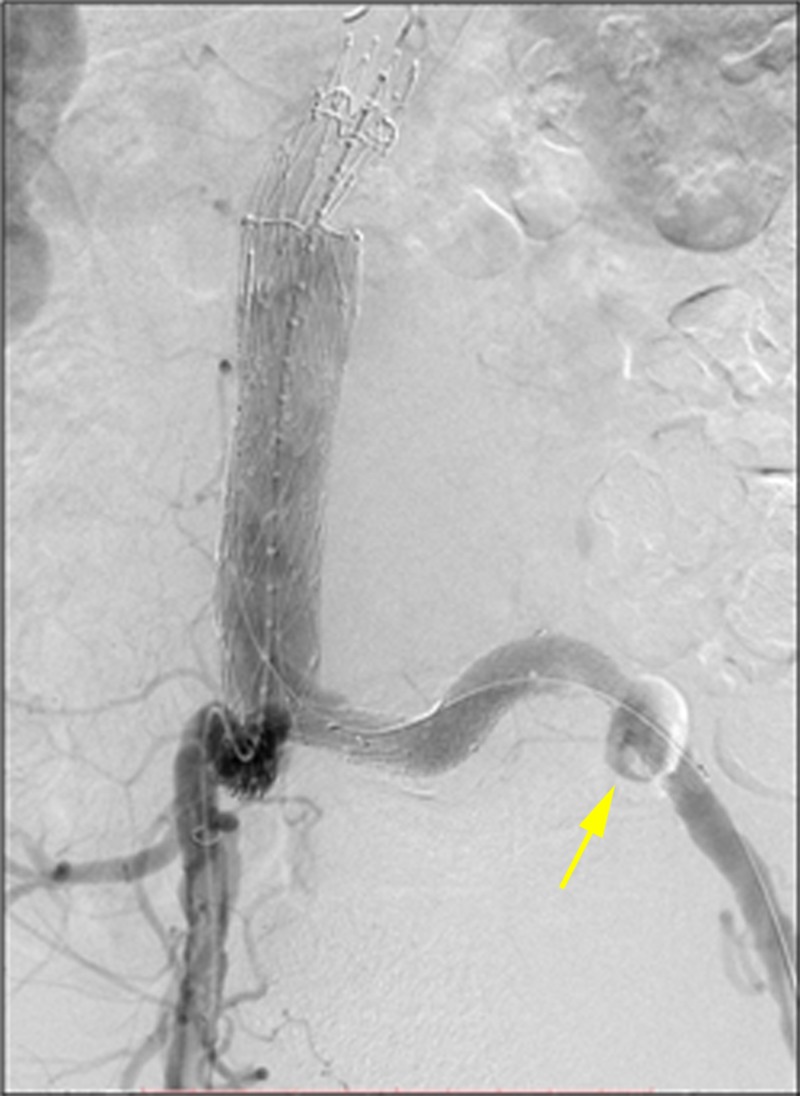
The final aortogram showed no endoleak after deployment of the Ovation limb extension into the external iliac artery. The Amplatzer vascular plug II (AVP II) is visualized (yellow arrow).

Following the deployment of the main body and proximal cuffs, a repeat aortogram was performed (Figure 10). A type IB endoleak was seen in the distal left common iliac artery; however, there was no leak proximally (Figure 11). Thus, an additional 14 mm x 100 mm Ovation iX iliac limb extension was deployed into the left external iliac artery and both iliac limbs were balloon dilated. A final aortogram demonstrated no residual endoleak (Figure 12). The wires and catheters were then removed, and hemostasis was obtained by securing the Pro-glide sutures, as well as brief manual pressure. Hemostasis was gained immediately. Protamine was not necessary. No intra- or post-procedural complications were encountered. 

**Figure 13:**
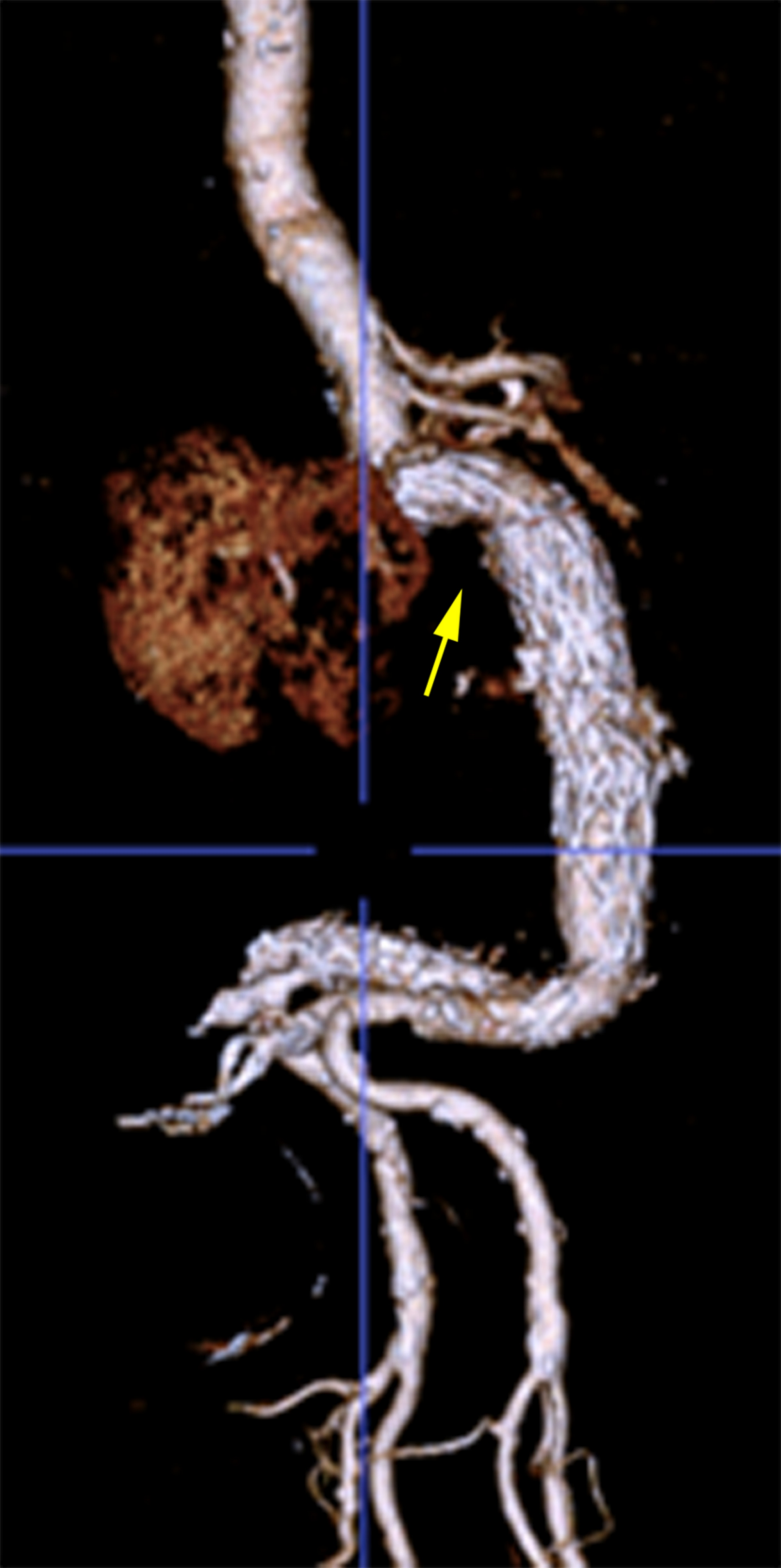
One-year follow-up CTA 3D reconstruction (sagittal orientation) showing a 90-degree neck angle and an intact stent graft CTA: computed tomography angiography; 3D: three-dimensional

**Figure 14:**
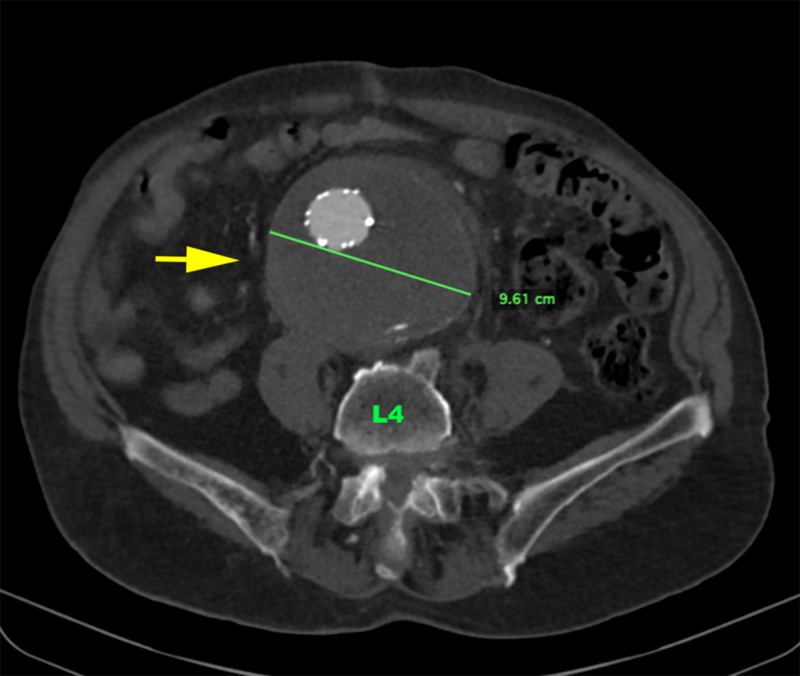
One-year follow-up CTA abdomen and pelvis axial image (level of L4) showing excluded AAA, which is stable in size at 9.6 cm (yellow arrow) AAA: abdominal aortic aneurysm; CTA: computed tomography angiography

At the one-month follow-up, the patient was asymptomatic, and the aortic stent-graph was structurally intact with no evidence of abdominal aortic aneurysm enlargement on imaging. Approximately 13 months post-procedure, the patient remained asymptomatic and underwent routine surveillance computed tomography angiogram (CTA) (Figure 13). The study showed that the aneurysm size remained stable at 9.6 cm with a type 2A endoleak originating from the right L3 lumbar artery (Figure 14). Endoleak treatment was considered; however, the patient and his family elected to continue with conservative management.

## Discussion

AAA has a prevalence of up to 8% in men over the age of 50 and an estimated 1,000,000 individuals living in the United States [8-9]. However, the majority of detected AAAs are small (< 4.0 cm) and those measuring > 5.5 cm account for less than 0.4% of all discovered AAAs [10]. EVAR is a minimally invasive procedure that has shown decreased mortality, length of intensive care requirements, and overall hospital stay compared to open repair in acute symptomatic AAA patients [2, 11]. EVAR historically involves surgical femoral artery cutdown and post-procedure repair due to the large profile of stent-graft delivery devices. However, a more recent procedural technique, known as percutaneous endovascular aneurysm repair (PEVAR), utilizes a pre-close technique facilitated by low-profile stent graft delivery systems and has demonstrated a lower incidence of vascular access site complications and decreased procedure time [12-14].

The feasibility of EVAR depends on the anatomical characteristics of the aneurysm. Initially, the provider should consider the diameter and tortuosity of the iliac arteries, which are required for device access. Next, the diameter of the aneurysm should be considered, along with the aneurysm neck angle. The aneurysm neck is the segment of the aorta which is between the proximal aspect of the aneurysm and the lowest renal artery. The description of the aneurysm neck is the most important factor in successful EVAR because it is the seal zone, which is crucial to prevent endoleak and distal migration of the stent graft. The neck of an AAA is described as “hostile” when it has unfavorable features. Aneurysm features which comprise a hostile neck include, along with their associated complications, a diameter > 30 mm (migration), neck length < 15 mm (type 1 endoleak), mural thrombus > 90 degrees (type 1 endoleak), mural calcification > 90 degrees (migration), and neck angulation > 60 degrees (migration and type 1 endoleak) - the latter of which is the most important determinant for failure of the endovascular repair [3-5]. Failure of endovascular repair is defined as an inability to completely exclude the AAA from the systemic circulation secondary to an inadequate seal between the stent graft and the native aorta wall [13].

Treatment of AAA with EVAR is well-documented. However, guidelines on the management of symptomatic very large AAAs (≥ 6 cm) with concomitant severe neck angulation (> 60 degrees) is an area of the current debate. Upon review of current literature, this case represents the largest unruptured symptomatic AAA with complex anatomical features to ever be successfully repaired via a completely percutaneous traditional endovascular technique to date [4]. This may be due to the large diameter and manufacturer characteristics of stent-grafts historically used in AAA repair.

## Conclusions

In our case, PEVAR was a viable option to treat this symptomatic AAA, despite multiple complex anatomical factors, as the device size did not require a surgical cutdown and iliac access vessels were of sufficient diameter. The authors believe that the confirmation of aneurysm stability at 13 months provides additional support for the ability to treat patients with symptomatic AAAs with minimally invasive techniques, such as PEVAR. Future studies that evaluate long-term outcomes of these large symptomatic AAAs with severe neck angulation treated with PEVAR are recommended.

Symptomatic abdominal aortic aneurysms should be admitted for observation and evaluated for potential candidacy of endovascular repair as these patients have a high risk of an impending rupture. Aortoiliac anatomy predicts successful outcomes of EVAR, the most important of which is the neck angle. PEVAR allows for decreased procedure time compared to EVAR with surgical access cutdown.

## References

[1] Wanhainen A (2008). How to define an abdominal aortic aneurysm--influence on epidemiology and clinical practice. Scand J Surg.

[2] Sadat U, Boyle JR, Walsh SR, Tang T, Varty K, Hayes PD (2008). Endovascular vs open repair of acute abdominal aortic aneurysms--a systematic review and meta-analysis. J Vasc Surg.

[3] Volpe P, Massara M, Alberti A (2017). Preliminary results of Aorfix™ stent graft to treat infrarenal abdominal aortic aneurysms with severe proximal aortic neck angulation. Ann Vasc Surg.

[4] Massara M, Prunella R, Gerardi P, De Caridi G, Serra R, Notarstefano S, Impedovo G (2017). A combination of thoracic and abdominal stent-grafts to treat an abdominal aortic aneurysm with hostile proximal neck. Ann Vasc Surg.

[5] Bastos Gonçalves F, de Vries JP, van Keulen JW, Dekker H, Moll FL, van Herwaarden JA, Verhagen HJ (2011). Severe proximal aneurysm neck angulation: early results using the Endurant stentgraft system. Eur J Vasc Endovasc Surg.

[6] Haug ES, Romundstad P, Aadahl P, Myhre HO (2004). Emergency non-ruptured abdominal aortic aneurysm. Eur J Vasc Endovasc Surg.

[7] Sullivan CA, Rohrer MJ, Cutler BS (1990). Clinical management of the symptomatic but unruptured abdominal aortic aneurysm. J Vasc Surg.

[8] Boll APM, Verbeek ALM, van de Lisdonk EH, van der Vliet JA (1998). High prevalence of abdominal aortic aneurysm in a primary care screening programme. Br J Surg.

[9] Mussa FF (2015). Screening for abdominal aortic aneurysm. J Vasc Surg.

[10] Von Allmen RS, Powell JT (2012). The management of ruptured abdominal aortic aneurysms: screening for abdominal aortic aneurysm and incidence of rupture. J Cardiovasc Surg (Torino).

[11] Krajcer Z, Matos JM (2013). Totally percutaneous endovascular abdominal aortic aneurysm repair: 30-day results from the independent access-site closure study of the PEVAR trial. Tex Heart Inst J.

[12] Gimzewska M, Jackson AI, Yeoh SE, Clarke M (2017). Totally percutaneous versus surgical cut-down femoral artery access for elective bifurcated abdominal endovascular aneurysm repair. Cochrane Database Syst Rev.

[13] Kim TH, Jang HJ, Choi YJ, Lee CK, Kwon SW, Shim WH (2017). Kilt technique as an angle modification method for endovascular repair of abdominal aortic aneurysm with severe neck angle. Ann Thorac Cardiovasc Surg.

[14] Penović S, Cambj-Sapunar L, Batinić T (2018). Endovascular repair of symptomatic (non-ruptured) abdominal aortic aneurysm in the University Hospital Centre Split. J Pharm Pharmacol.

